# Young people’s attitudes towards online self-help single-session interventions: findings from a co-produced qualitative study

**DOI:** 10.1186/s40359-025-02727-8

**Published:** 2025-04-24

**Authors:** N. Higson-Sweeney, S. Dallison, E. Craddock, B. Teague, C. Payne-Cook, J. Leas, A. V. Slastikova, H. Peel, L. Biddle, M. E. Loades

**Affiliations:** 1 Department of Psychology, University of Bath, Claverton Down, Bath, UK; 2 Department of Experimental Psychology, University of Oxford, Oxford, UK; 3 NSFT Research, Norfolk and Suffolk NHS Foundation Trust, Norwich, UK; 4 Department of Clinical Psychology and Psychological Sciences, University of East Anglia, Norwich, UK; 5 Population Health Sciences, Bristol Medical School, University of Bristol, Bristol, UK

**Keywords:** Participatory research, Mental health, Online help-seeking, Qualitative study, Single-session interventions, Youth

## Abstract

**Background:**

Many young people experience at least subthreshold depression symptoms which impact their functioning. Yet, access to evidence-based help is limited, with barriers such as service thresholds, stigma, and lack of knowledge about mental health and available services. One way to ensure young people have access to free, early, immediate and anonymous help is through online self-help single-session interventions. This study aimed to qualitatively explore young people’s perceptions of and attitudes towards these interventions.

**Methods:**

Twenty-four young people (UK based, age 15–18) took part in qualitative semi-structured interviews which were hosted online and co-conducted with a young research team (*N* = 4, age 16–18), during which we described online single-session interventions and asked participants for their perspectives. Together with our young researchers, we analyzed the data using reflexive thematic analysis.

**Results:**

Three themes were generated: (1) Will it help, or won’t it? Hope versus skepticism; (2) Why this approach? Benefits of single-session interventions for young people; and (3) Have you considered this? Logistics for implementation.

**Conclusions:**

The current study highlights that whilst young people perceived there to be many benefits associated with online single-session interventions, including anonymity, easy access, and lack of disclosure, they expressed doubts regarding sufficiency and ability to address severe mental health problems. Despite this, the potentially preventative effects during the early stages of help-seeking were highlighted, alongside single-session interventions acting as a gateway to further help-seeking and support. However, logistical considerations should also be reflected upon when developing online single-session interventions, including where they are advertised, age appropriateness, and how to demonstrate trustworthiness.

**Supplementary Information:**

The online version contains supplementary material available at 10.1186/s40359-025-02727-8.

## Background

Youth mental health is an urgent public health issue. Globally, a meta-analysis of studies published between 2001 and 2020 found that the prevalence of self-reported elevated depressive symptoms among 10–19 year old’s was 34%, with one-year and lifetime prevalence rates for major depressive disorder of 8% and 19%, respectively [1]. Depression unfavorably impacts young people’s (YP’s) functioning, including school attendance [2], academic attainment [3], social life [4], and longer-term mental health outcomes [5, 6]. Even subthreshold symptoms of depression that are clinically relevant but do not yet meet full diagnostic criteria are associated with higher suicidality and lower social and family functioning [7].

Evidence-based treatments exist for youth mental health but are hard to access. These include psychological therapies [8], such as Cognitive Behavioral Therapy (CBT), which is widely recommended in treatment guidelines for anxiety and depression [9–11]. However, economic pressures and the lack of trained providers limits the availability of these treatment options and specialist mental health care [12]. Child and Adolescent Mental Health Services (CAMHS) referral threshold criteria mean that those YP who do not present with risky behaviors and/or severe functional impairment are less likely to access or receive help [13, 14]. Furthermore, even if they do meet the threshold for help, they likely have to wait to access it [15]. These service-level barriers, combined with other internalized barriers such as stigma, lack of knowledge about mental health problems and help-seeking options, and a preference for being autonomous [16, 17], mean that there are a considerable number of YP who are struggling with their mental health, but are unable to seek support for it. This makes it imperative to develop effective treatments that can be offered at scale in a timely manner.

Digital mental health interventions (DMHIs), including those designed to be used independently as self-help, are a more scalable means of providing evidence-based treatment and may bypass various help-seeking barriers that YP may face. However, many DMHIs, such as internet-based CBT (iCBT), are designed in the same way as traditional clinic-based treatments; they are built as a course of treatment, assuming repeated use over time to accrue the benefits [18]. However, in practice, many YP discontinue use after one or two sessions, especially for self-help digital treatments [19, 20]. Furthermore, DMHIs like mental health apps may have associated costs and device compatibility requirements, which create further barriers to access [21].

Single-session interventions (SSIs) represent a potential solution to the problem [22]. SSIs are designed as one-off therapeutic interventions, avoiding the need for repeat usage. A meta-analysis of SSIs involving 10,508 adolescents with mental health problems found a pooled mean pre-post intervention effect size of *g* = 0.32 [23]. Although relatively small, these effects are noteworthy, given their brief treatment duration, and more extensive therapies do not significantly outperform SSIs. Notably, self-help SSIs were also found to be as effective as therapist-delivered SSIs [23].

Online self-help SSIs that can be accessed anonymously can be effective in reducing mental health symptoms and may better reach stigmatized individuals. This is evidenced by several brief (< 30 min) SSIs developed by the Lab for Scalable Mental Health in the United States, which include psychoeducational materials, videos, vignettes, and self-reflexive exercises. The two SSIs developed by this group with the strongest evidence for reducing depressive symptoms in American adolescents are cognitive and behavioral. The cognitive SSI [24] focuses on the malleability of traits or habits (growth mindset), while the behavioral SSI [25] emphasizes that engaging in valued activities can enhance positive mood (behavioral activation). Three randomized controlled trials found these SSIs effective at reducing depression symptoms up to a 9-month follow-up, even during the pandemic [26–29].

Findings predominantly from the Lab for Scalable Mental Health indicate that these online SSIs, beyond trial use, could be valuable as openly accessible resources for YP struggling with their mental health. These SSIs have been found to be acceptable and useful for diverse YP, including sexual and gender minority YP and ethnic minority YP [28–30], and have also been successfully translated, culturally adapted, and disseminated in low-resourced and minoritized communities, with evidence of good uptake, acceptability, and utility [31].

However, YP’s perceptions of online self-help SSIs have mainly been investigated by seeking feedback on acceptability from those who have already consented into a study offering SSIs and completed an intervention. These studies may not capture the views of YP more broadly, some of whom may or may not be interested in starting or completing an SSI. These existing studies have also generally depended on quantitative data from Likert scales on feedback questionnaires, complimented by free-text box written comments. Whilst the latter does provide some qualitative feedback, a more in-depth exploration of YP’s perceptions of online self-help SSIs would help to further our understanding of how these resources are perceived and experienced when they are first introduced to YP, as well as their likely uptake. To ensure that the SSIs being developed are useful and relevant to the target population, it is imperative that this gap in our knowledge is addressed. As such, this study aimed to qualitatively explore YP’s perceptions of and attitudes towards online self-help SSIs, with a focus on understanding the benefits and limitations of this treatment approach in the context of public health.

## Methods

For this qualitative study, we conducted individual semi-structured interviews. These were co-conducted by pairs of researchers, one experienced researcher from Norfolk and Suffolk NHS Trust (NSFT) and one member of our Young Research Team (YRT). The YRT comprised four high school students aged 16–18 years old, who underwent training for data collection and analysis, and were compensated for their time. Our collaboration with the YRT was guided by a participatory framework approach and co-production, where individuals who are directly affected by the topic under study are actively involved in the research process as equal contributors alongside researchers and other stakeholders [32, 33]. In the context of the current study, co-production involved the active engagement and involvement of YP from the earliest possible point in the research process through to dissemination, with the YRT collaborating with researchers to conduct the interviews, analyze the data, write research papers, and develop infographics of the findings. This participatory approach was beneficial, as this is an underexplored research area and the YRT’s involvement ensured that the interview questions were age-appropriate and easy to understand; that the generated themes were meaningful and reflected the nuance of YP’s insights; that the research papers were written clearly; and that the findings would be accessible to a broader audience, including other YP. For a more comprehensive explanation regarding the YRT and the methods for this study, please see [34]. A reflective summary from the perspective of the research team is also currently under review.

### Participants

English-speaking adolescents aged 13–18 residing in the UK were eligible to take part in this study, regardless of previous help-seeking experience or self-report depressive symptoms. Recruitment took place during July-August 2023. Adverts were collaboratively developed with input from YP advisors to ensure visual appeal, ease of understanding, and applicability to the target sample. Recruitment was carried out through social media platforms such as Instagram and X (formerly Twitter), as well as mailing lists from schools and community organizations targeting diverse groups. We utilized convenience sampling, contacting all interested participants who completed an online form after passing bot checks. Sample size was guided by the principle of information power [35], which suggests that the more information power a sample holds, the fewer participants needed to collect enough data to sufficiently addresses the research questions. Information power is an alternative approach to data saturation, which originated in the context of grounded theory and implies that it is possible to reach a point where no new insights are generated [36]. Instead, through information power, recruitment ceased once the research team deemed that sufficient data had been collected to comprehensively address the study research questions.

## Measures and materials

Participants self-reported demographic information (e.g., age, gender identity) and completed the Patient Health Questionnaire-2 (PHQ-2) [37] on Qualtrics at the beginning of the interview. A flexible semi-structured topic guide was used to guide the interviews, which was developed with input from the YRT. The interview was divided into four parts. The first part focused on perceptions and attitudes towards online help-seeking for mental health support, using a persona called *‘Sally’* (see Figure S1 in supplementary materials) to prompt reflection and discussion. Sally was initially developed as a teaching resource in collaboration with a young adult with lived experience to describe a young person with subthreshold symptoms of depression. Her symptoms were designed to reflect criteria from the fifth edition of the Diagnostic and Statistical Manuel of Mental Disorders (DSM-5) [38] as well as YP’s lived experience accounts of depression [39, 40]. Sally’s demographic characteristics included consideration of known vulnerability factors for developing depression, such as being female. In the current study, we adapted this teaching resource with the YRT by simplifying the language used.

The second and third parts of the interview used screen sharing and ‘think aloud’ techniques to elucidate participants’ decision-making processes when searching for support online, and their thoughts regarding a selection of pre-existing websites (e.g., Young Minds) and adverts (e.g., Lab for Scalable Mental Health). The fourth and final part of the interview described online SSIs and included questions and probes designed to elicit participants’ thoughts and attitudes towards their use and design (see Figure S2). The current paper focuses on data collected from parts three and four of the interview; data from parts one, two and three are explored in [34].

### Procedure

Ethical approval was obtained from the University of Bath Psychology Research Ethics Committee (23–057) and the study protocol was pre-registered on the Open Science Framework in June 2023 (https://osf.io/a6k7r). Interested participants accessed detailed study information through a link/QR code to a Qualtrics survey where they also completed an online screening form to confirm eligibility based on age and place of residence. Written consent (assent and parental consent for under 16’s) procedures followed, with participants providing contact details and generating a pseudonym for documentation and reporting purposes. Interviews were conducted online via Microsoft Teams, with interviewers and participants keeping their cameras on throughout. Interviews ranged from 23 min to 1 h 13 min (*M* = 42 min, *SD* = 11 min), with roughly an equal amount of time spent on each of the four parts of the topic guide. Participants were compensated with a £20 Amazon e-voucher for their time and effort.

### Data analysis

Transcripts generated through Microsoft Teams were checked and corrected for accuracy and anonymized prior to analysis. We used pseudonyms generated by the participants themselves. Descriptive analysis of the demographic and clinical characteristics of the sample was performed using Microsoft Excel.

Reflexive thematic analysis [41, 42] was employed to iteratively code the data and generate themes, from a data-driven perspective. The analysis followed Braun and Clarke’s six-phase model, beginning with phase one, where transcripts were divided between three members of the research team and one young researcher (NH-S, CPC, EC, AS), who familiarized themselves with the data through reading, re-reading, and taking initial notes of interest. In phase two, transcripts were openly double-coded by two groups (group 1: NH-S and CPC; group 2: EC and AS), which was done at both a semantic and latent level. All codes were then integrated and refined by SD to identify overlap between coders, where similar sentiments may have been phrased in slightly different ways. A final list of codes was created, which was used in phases three and four by NH-S and SD to generate and refine initial themes and subthemes. In phase five, these themes and subthemes were shared with the wider team for continued refinement and defining. In phase six, NH-S, SD, EC and MEL wrote up the final analysis, with feedback from the wider authorship team.

Content analysis was also used to allow for the quantification of suggested SSI content [43]. This was led by NH-S, and involved repeatedly reading the data for immersion, before combing through it to identify relevant words and phrases of interest. These words and phrases were transformed into codes, which were then refined into categories of related meaning.

Further, the findings from the study were retrospectively mapped to the Consolidated Framework for Implementation Research (CFIR), which is a comprehensive framework that identifies key factors influencing the implementation of interventions. The CFIR was originally published in 2009 [44] and subsequently updated in 2022 [45] with five domains of assessment: intervention characteristics; outer settings; inner settings; individual characteristics; and implementation processes. The CFIR incorporates constructs from multiple established implementation models and frameworks, thus offering a comprehensive structure for examining the complex factors that influence implementation outcomes. The CFIR is important for evaluating public health interventions as it helps identify barriers and facilitators, guiding strategies to improve adoption, scalability, and sustainability. As findings from the current study will be used to develop future SSIs, this retrospective mapping was led by MEL. Here, it is important to note that the CFIR is a flexible framework that can be used prospectively and retrospectively, and whilst prospective use may have allowed for data collection to be deductively guided by the five domains, resulting in a better fit of findings, this is an under-explored research area that required an inductive approach. As such, retrospective mapping was deemed to be the most appropriate method for utilising the CFIR. MEL systematically coded the themes and subthemes we had generated from our qualitative data against the CFIR domains. These were then reviewed and refined with input from the wider research team, particularly LB.

Throughout the study, we worked within a contextual, critical realist perspective, considering our findings to be reflections of an underlying reality whilst simultaneously recognizing the influence of social and contextual factors on both participant experiences and our interpretations as researchers.

The research team, including a trained clinical psychologist specializing in adolescent depression, had a collective interest in YP's mental health. The team includes members with diverse expertise and perspectives, contributing to a comprehensive understanding of the study findings.

## Results

Twenty-four YP participated in the current study, with some diversity in gender identity and ethnicity (see Table 1 for participant demographics). The mean PHQ-2 score for the sample was 2.29. As the cut-off for the presence of depression is 3, this suggests that the sample predominantly exhibited subthreshold symptoms; however, individual scores ranged from 0 to 6, indicating a range of experienced depression severity [37].

**Fig. 1:**
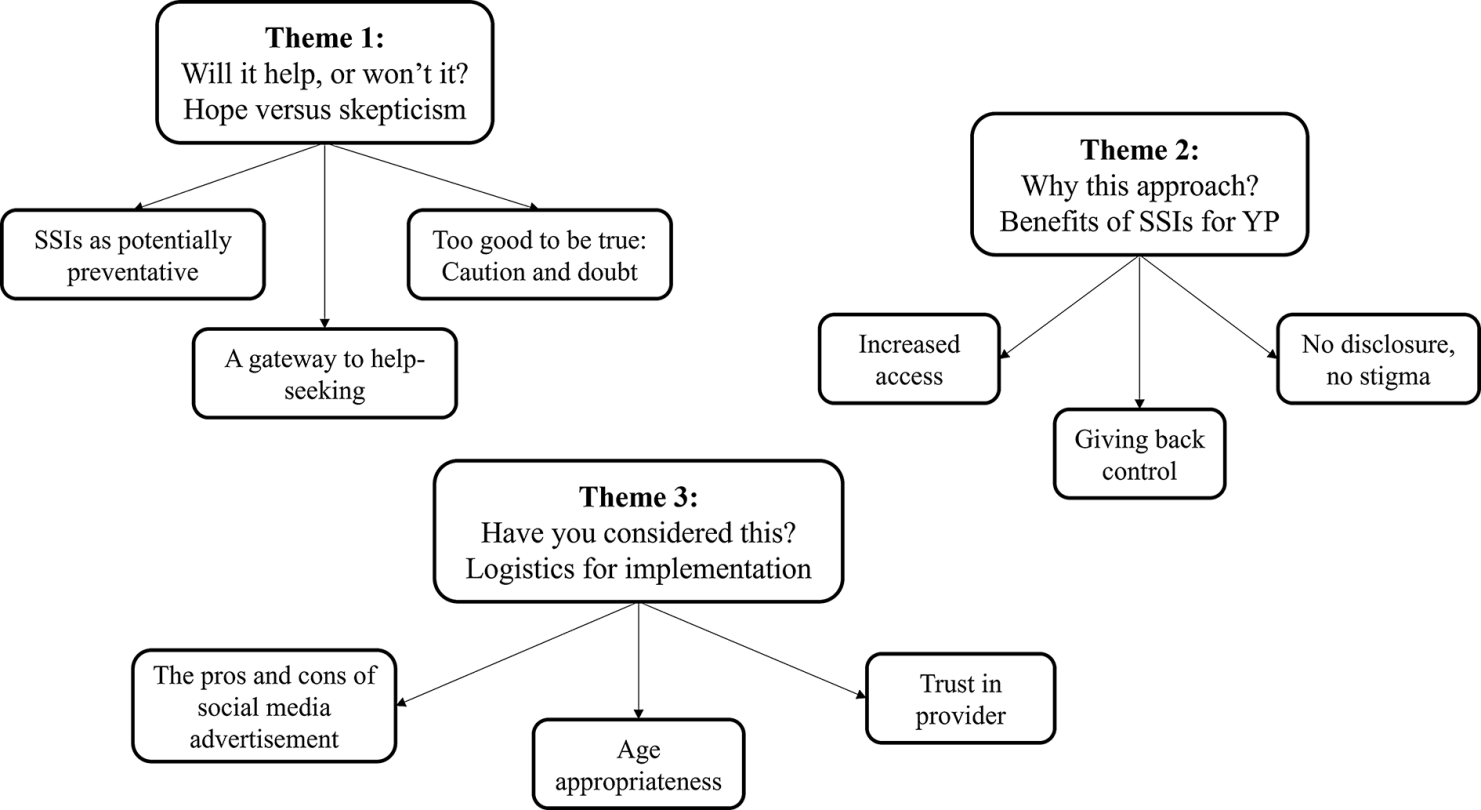
Thematic map

Our data analysis process generated three themes which collectively showcase YP’s thoughts and attitudes towards the use and design of online self-help SSIs: (1) Will it help, or won’t it? Hope versus skepticism; (2) Why this approach? Benefits of SSIs for YP; and (3) Have you considered this? Logistics for implementation (see Fig. 1 for a thematic map).

**Table 1:** Participant demographics

Characteristic	Response options		*N*
Age (years)	15–16		6
	17–18		18
Biological sex at birth	Female		17
	Male		5
	Prefer not to say		2
Gender identity	Woman/girl		16
	Man/boy		4
	Genderqueer		1
	Male-to-female transgender		1
	Agender		1
	Non-binary		1
Ethnic group	White	9 English/Welsh/Scottish/Northern Irish/British 2 Other (Russian/Ukrainian; Jewish)	11
	Asian/Asian British	1 Bangladeshi 1 Indian 2 Other (Taiwanese; Afghan)	4
	Black/African/Caribbean/Black British	6 African 1 Other (no further information given)	7
	Mixed/Multiple	1 White and Black African 1 White and Black Caribbean	2
Depression severity (PHQ-2)		*M* = 2.29 (*SD* = 1.73), range = 0–6	

Note M = Mean; N = Total number of participants; PHQ-2 = Patient Health Questionnaire-2

### Theme 1: will it help, or won’t it? Hope versus skepticism

Whilst YP often spoke positively about the potential of online self-help SSIs, there was an undercurrent of skepticism throughout the interviews, with participants doubting the ability of an SSI to sufficiently meet YP’s needs. This tension is explored through three subthemes.

### SSIs as potentially preventative

YP were quick to describe the potential benefits that a one-off SSI could afford when discussing the persona of *‘Sally’*. They particularly emphasized how SSIs could provide validation, teach new coping mechanisms, and prevent the need for further, more intensive support; *“she may get the information that she needs in the single session”* (Ami).

“I think it can be really beneficial for her in the end, especially if it is as good as it sounds. I think she could come out feeling a lot more positive about herself.” (Aco).

Within this seemed to be a ‘nothing to lose’ mentality, with several YP expressing the sentiment that *“if you think that problem can be dealt with without having to [go to the] GP, I feel like it’s better than getting no help at all”* (Alex). As SSIs are designed to be relatively short, YP generally seemed to feel that the minimal time commitment was worth investing in something that may be helpful.

### A gateway to help-seeking

Participants acknowledged that the thought of seeking in-person support could feel *“quite daunting”* (Sophie). As such, SSIs were seen as *“a good starting point”* (Maisie) in the help-seeking process by providing YP with psychoeducation and insight into what they might be experiencing in an accessible, confidential way. YP reported feeling that “*even having a little bit of knowledge […] can go a long way in the long run”* (Alex). Subsequently, participants also described how SSIs could act as *“a stepping stone”* (Zigzag) to further mental health support, by providing YP with the language and information they need to approach other people.

“I feel like it [could] be quite a good resource if [*‘Sally’*] wanted to try something first, like, before she was comfortable enough to go to friends and family and counsellors and stuff.” (Luxseal).

### Too good to be true: caution and doubt

However, YP expressed some reservations about SSIs, particularly the ability of a single session to meet individual needs. For some participants, this concern related to the lack of direct contact and relationship-building with a mental health professional, and the absence of in-person communication.

“If I wasn’t talking to somebody directly, I’m unlikely to join. I’m not that type of person. I prefer speaking to people.” (John).

Others had concerns about whether an SSI could sufficiently help YP with more severe mental health problems. In contrast to the ‘nothing to lose’ mentality highlighted in the first subtheme, for these YP, chancing an SSI they had no confidence in did not seem worthwhile.

“If I was feeling hopeless, I probably wouldn’t think a single session […] that’s not going to work […] just something as simple as an online session […] it’s not going to be worth my time.” (Matilda).

Some of this concern seemed tied to the fact that SSIs often focus on self-help and require an element of proactivity in seeking out and engaging with the support provided. Depending on the severity of experienced symptoms, some participants reported that this could act as a barrier to SSIs, and that researchers should keep this in mind when developing content.

“To take responsibility of your own healing again is very difficult when you’re-when-when you’re feeling very hopeless.” (Maisie).

Generally, participants seemed more comfortable and accepting of SSIs if they were not presented to YP as an instant fix, and that emphasis was placed on treatment as a process.

“I think it just needs to be less about you do this once and you feel fabulous and needs to be more it’s slow steps and by integrating this into your, like, routine or doing this can help you feel a bit better instead of making it sound like a magical cure.” (Sophie).

### Theme 2: why this approach? Benefits of SSIs for YP

Participants discussed some of the potential benefits that online self-help SSIs can provide to YP, focusing on the ways in which elements of SSIs may actively combat barriers faced through more traditional help-seeking routes. These are discussed across three subthemes.

### Increased access

YP spoke positively about the accessibility of SSIs: “*I guess because it’s free*,* anonymous*,* really easy to access um kind of like*,* why not*?” (Rose). By accessibility, participants referred to the online format of SSIs, meaning that they can be accessed conveniently anywhere at any time, as well as the short duration and subsequent flexibility around their busy lives.

“It’s just there and it’s easy if you want to access it. And it’s also not in person, which is quite good because you can fit it in with your schedule. If you have a lot of clubs going on, if there’s a lot of schoolwork there to do, it’s quite easy to do.” (Flower).

YP also reflected on the importance of instant access to SSIs without having to wait, which was deemed to be a facilitator in increasing access to mental health support: “*Its online and [there’s] no waiting list*” (John). The absence of gatekeepers (i.e., parents, caregivers, healthcare professionals) in the process of accessing the SSI was also deemed to increase access for YP: “*it’s just something that [Sally] can click and do and it’s not a long wait for an email from this person*,* get your parent to sign it off*” (Aco).

### Giving back control

Participants discussed how some adults may believe that YP lack motivation when it comes to looking after their mental health, which they argued to be a misconception. Instead, they emphasized the opposite; that many YP actively want to take charge of their mental wellbeing but may feel unable to do so or feel disempowered.

“I think a lot of people just think teenagers aren’t trying […] there’s a kind of rhetoric that we [are] lazy and decided to be depressed and all of this, but that’s not actually true.” (Maisie).

Participants reflected on how SSIs might provide YP with this sense of control and empowerment, giving them the tools to “*get them involved with their own mental health and supporting it*” (Gale) and *“the power to do it in their own time*” (Sophie). As SSIs are remotely accessed and not monitored, participants reported that YP would also not feel pressured to complete it, giving them continued control over their engagement; “*you can always click on it and be like oh this isn’t for me and click off that. It’s not like a commitment to anything*” (Rose).

### No disclosure, no stigma

Across interviews, participants consistently highlighted “*the fact that you don’t have to speak to anyone about it*” (Jago) as one of the most salient benefits of SSIs. YP liked that SSIs allow for anonymity and do not require disclosure at the point of access. Participants expressed that SSIs were a discrete way of accessing mental health support, which they considered as important given the potential stigma a young person might experience– or fear experiencing– from friends and family.

“You might be going through something and you fear maybe approaching a friend or your parent because they might spread it to someone else and you don’t want that kind of embarrassment or stigmatisation.” (Sabi).

Participants also considered how the lack of human contact in SSIs meant that YP are not required to disclose or discuss their experiences whilst still receiving mental health support. This was seen as particularly beneficial for YP like Sally who are seeking help for the first time and may struggle or not want to verbalize their thoughts and feelings.

“The idea of not having to explain yourself to a person cause often that’s quite difficult when you’re in that state of mind, you don’t always know how you’re feeling.” (Matilda).

### Theme 3: have you considered this? Logistics for implementation

Despite the perceived benefits of SSIs, participants reflected on several areas for consideration when designing, advertising, and implementing SSIs, which are encompassed by three subthemes.

### The pros and cons of social media advertisement

The idea of advertising and accessing SSIs online was met with a largely positive response. Social media was identified as something YP *“have access to and are on a significant proportion of the time”* (Maisie) and *“how most people get their information now”* (Sophie), making it an appropriate place to advertise interventions aimed at this population.

“Reaching out to young people on the platforms they already use, um, is important.” (Maisie).

That said, some YP expressed doubts about the level of engagement and interaction an SSI would receive if it was advertised on social media, with the understanding that YP use these platforms to *“just scroll through things”* (Zigzag) rather than actively seek mental health support.

“If they are out and about in the day and they’re just quickly scrolling through their Instagram or something and they see it, maybe it wouldn’t be something to click on.” (Matilda).

### Age appropriateness

YP emphasized the necessity of an SSI to be age appropriate, both in terms of its content and how it is advertised. When shown example adverts for an SSI, participants’ attitudes towards the use of color, illustrations, and wording elicited different responses as to whether they would engage in the intervention - although there was recognition throughout that *“different age ranges would want different information”* (Matilda), emphasizing that one size does not fit all YP. As an example, some YP said that they liked cartoon illustrations as it made the target audience of the SSI clear, whereas others described it as off-putting; *“I don’t think I’m able to get what I’m looking for with these pictures”* (Ami). Wording was also identified as particularly important, although there was a fine line between writing in a way that was understandable and accessible to YP, versus writing in a way that felt patronizing and demeaning.

“If I saw this I’d be like I’m 17, I don’t know this-this kind of language and advertising to me […] would put me in a worse mood because it made me feel like I’m more of a child again.” (Jago).

Age-appropriateness was also a concern from the context of included topics, with YP expressing that *“it couldn’t really be anything childish*,* because then they might be […] pushed away from it”* (Flower). The content analysis of what topics participants suggested they would want to see in an SSI targeted at YP can be seen in Table 2.

**Table 2:** Content analysis of suggested SSI topics

Topic	*N* (%)
Coping mechanisms and strategies (including healthy habit formation)	15 (62.5)
Developing self-compassion and improving self-esteem	9 (37.5)
Reflecting on feelings (including mindfulness)	6 (25)
Causes of low mood	6 (25)
Managing/overcoming depression	6 (25)
Validating messages (including lived experience perspectives)	5 (20.8)
Next steps (including disclosure and further signposting)	5 (20.8)
Anxiety	4 (16.7)
Stress (including school stress)	3 (12.5)
Self-care and general wellbeing	3 (12.5)
Psychoeducation about mental health	3 (12.5)
Managing relationships	3 (12.5)

Note N = number of participants who endorsed the topic; % = percentage out of 24 participants

### Trust in provider

The challenge of determining the authenticity of an SSI was frequently discussed and was usually based on who was providing it. Clear signposting within adverts to organizations that YP deemed reputable, such as universities, charities, and the NHS, increased both the credibility and trustworthiness of the intervention, and the likelihood of a young person engaging in it. Still, if the YP had not heard of that specific organization, an element of doubt would remain.

“It’s backed by a university so I would say it’s quite trustworthy. The only thing I would say is like I’ve never heard of that university in my life.” (Sophie).

In circumstances like these, YP explained the precautions and additional steps they would take to determine the trustworthiness of an SSI, such as Googling the institution.

“I probably want to find out a bit more about that before I like delve into it, but it’s I think obviously ‘cause it’s from a university, again I think it’s still quite credible.” (Toyosi).

### Mapping our themes against CFIR

**Fig. 2:**
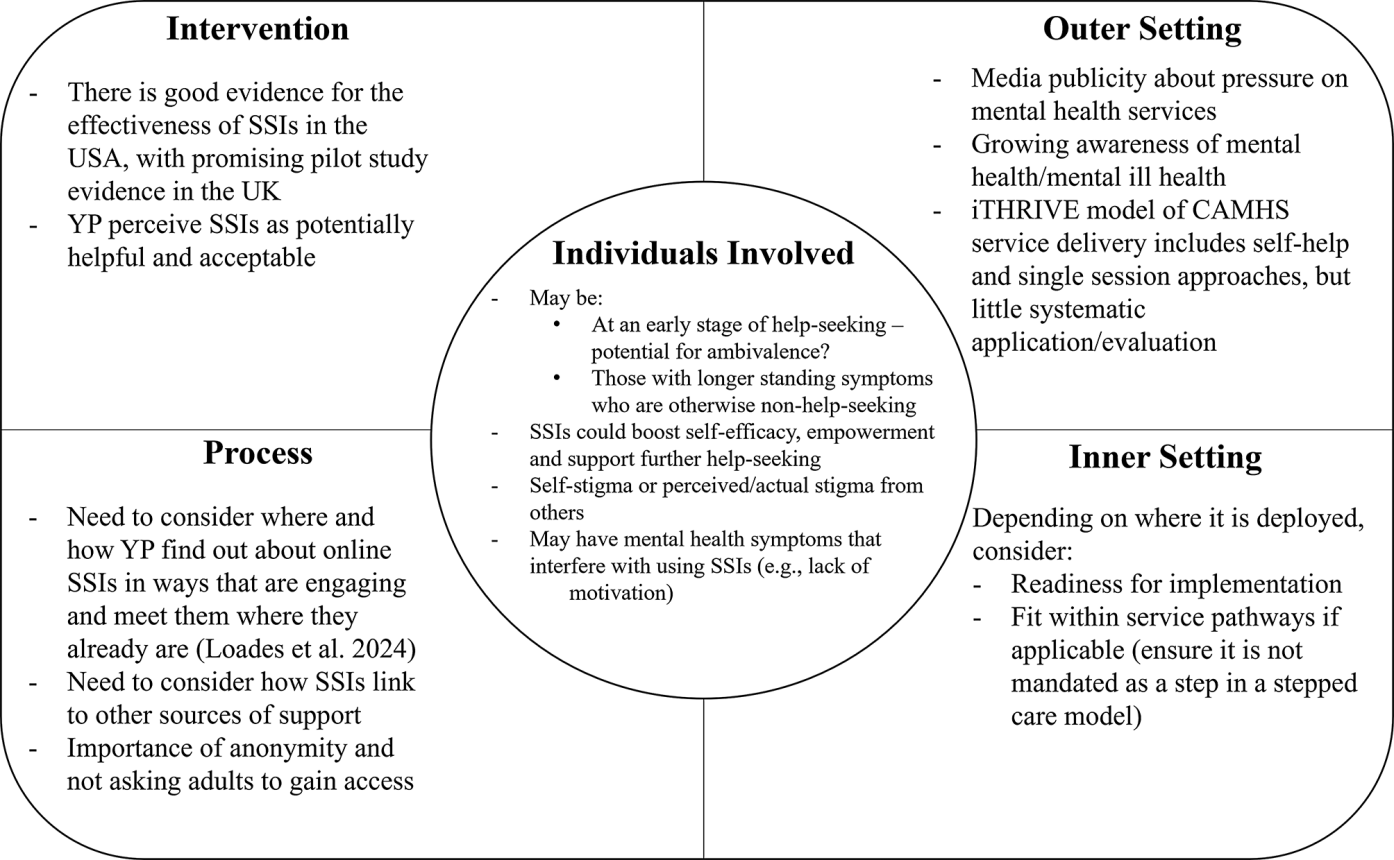
Conceptualization of implementation implications using the consolidated framework for intervention research (CFIR) model [45]. Note. CAMHS = Child and Adolescent Mental Health Services; SSIs = Single-Session Interventions; YP = Young People

Figure 2 illustrates the CFIR model mapping of implementation implications from our findings, highlighting factors such as media publicity, growing awareness of mental health, and the importance of anonymity, self-efficacy, and readiness for implementation in the context of SSIs.

## Discussion

Through qualitative interviews, our findings suggest that YP perceived SSIs to be potentially helpful and preventative when looking for early support for their mental health. Perceived benefits included immediate access without waiting, asking someone for help, or needing to be in a specific location. Additionally, SSIs were seen as empowering and providing YP with control, given their anonymity and the lack of need for disclosure. Participants highlighted several logistical considerations for implementing SSIs as a public health offering, including age appropriateness, where and how SSIs are advertised to make YP aware of them, and markers of trustworthiness and authenticity. However, there were concerns regarding sufficiency and the impact of symptom severity.

Across several subthemes, our participants highlighted that the perceived benefits of SSIs, including the lack of waiting lists or gatekeepers, mean that YP can receive immediate, in-the-moment support for their mental health. This is consistent with the purpose of online self-help SSIs, which should be seen as an expansion of current provision of early access interventions rather than a replacement for more intensive support [18], with affordances such as immediacy, anonymity, and remote access. Previous research has emphasised the importance YP place in immediacy, particularly in the context of crisis support [46], but also for help-seeking in general [47]; SSIs seem able to meet this need.

Additionally, online SSIs were seen by participants as offering an opportunity for those earlier in the help-seeking process to receive support, which also aligns with intended value [18]. Specifically, in the sixth subtheme, they mentioned that self-help SSIs may be particularly useful for those who do not feel comfortable verbalising their feelings to get help, making them more inclusive. This mirrors quantitative user experience feedback from those who have completed SSIs in the USA [27–29, 48–50] and UK [51], as well as qualitative user experience feedback from YP in a UK pilot of an online SSI in a paediatric health context, who indicated that it was a potential first step in overcoming their difficulties [52]. The current study adds weight to these prior findings by going beyond acceptability, and including the views of YP who have not previously engaged with SSIs.

**Fig. 3:**
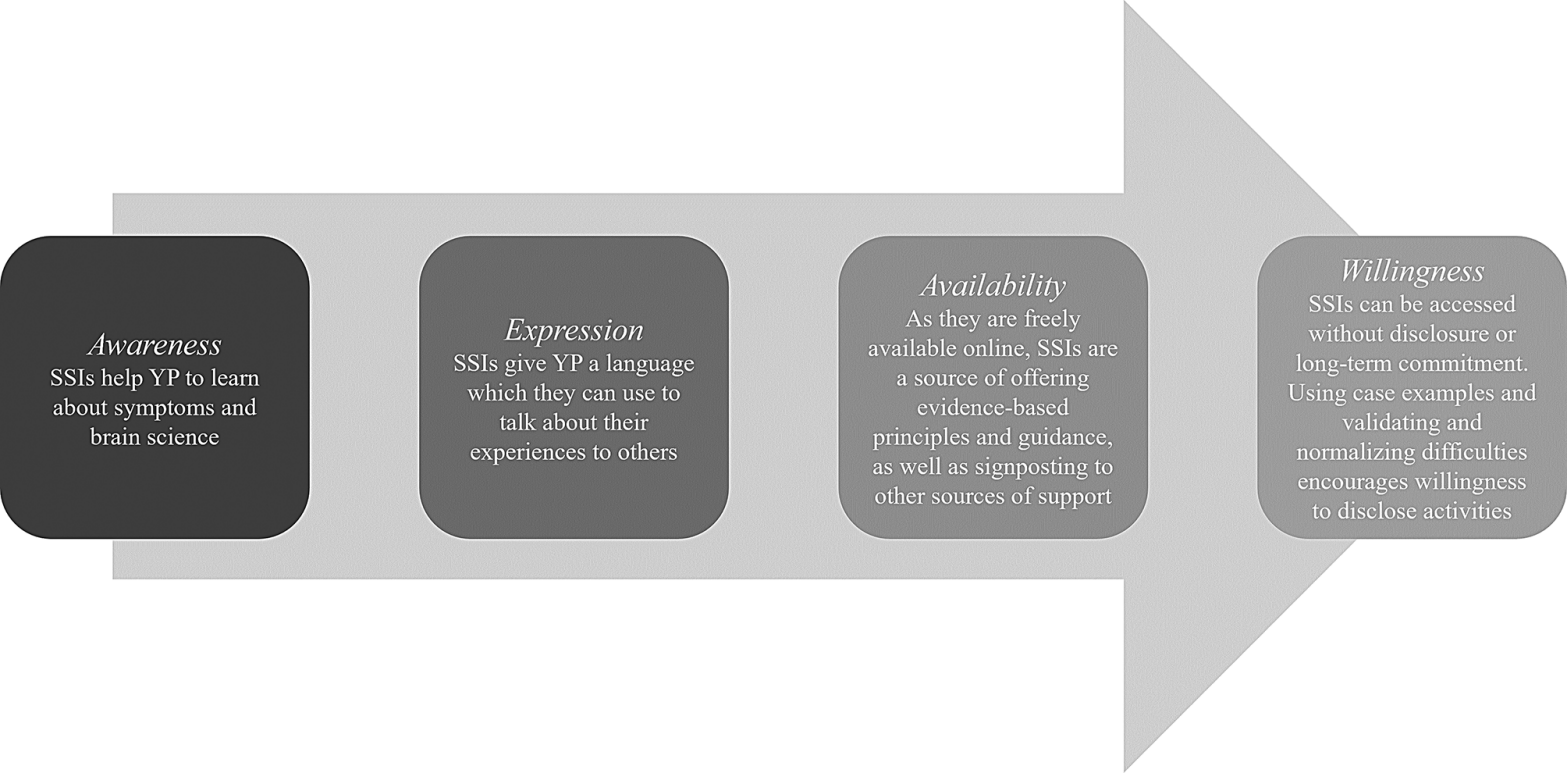
How online self-help SSIs might aid the steps of the help-seeking process described by Rickwood [53].Note. SSIs = Single-Session Interventions; YP = Young People

However, in the third subtheme, participants highlighted concerns that SSIs might not be sufficient as a standalone treatment. This is where the role of SSIs as a potential gateway to further help-seeking is emphasised. Help-seeking can be understood as a process; for example, Rickwood’s help-seeking model starts with problem recognition, followed by recognition of the need for help, expressions of help, the availability of sources of help, and the willingness to seek these out and use them [53]. Seeking help online may be an accessible, anonymous, and less pressurized alternative for individuals facing mental health issues [47], and can be seen to mitigate some of the potential obstacles identified in Rickwood’s model. For example, accessing online SSIs does not necessarily require problem recognition beforehand, particularly if positioned as a public health support and advertised using terms like ‘wellbeing’ rather than diagnostic labels. Further, online SSIs do not require YP to commit to disclosing to a professional, which is often a defining moment within the help-seeking process and can be a contributor to the cycle of avoidance [54] (see Fig. 3 for further examples).

However, for YP to access SSIs, we first need to consider how we let them know about the availability of these resources. Currently, it seems pertinent to advertise SSIs in places that YP already spend time and may look to for help, including schools and online spaces, such as social media [55]. This was largely supported by participants in the current study, who felt that YP’s widespread use of social media made it an appropriate place to advertise. However, there was also the acknowledgement that YP may not explicitly use social media for help-seeking, which could decrease engagement. This makes it important to also advertise the availability of SSIs to YP’s supporters, such as parents and caregivers, alongside frontline professionals who may be the first to notice YP struggling, like teachers and General Practitioners [56]. Other places that YP already look for help, such as moderated forums and mental health apps, as well as anonymous online support services like Kooth and Shout in the UK, could also be leveraged to signpost to SSIs as an addition to their existing offerings.

A particular aspect that our participants highlighted, which is central in the current context of misinformation online, is that of trustworthiness and credibility. Interestingly, a recent study in routine clinical care in the USA found that the credibility and trustworthiness of DMHIs was more of a concern for providers than service users, who focused more on usability and accessibility [57]. However, perhaps due to generational differences, with Generation Z having grown up as ‘digital natives’ [58], our participants were also concerned about trustworthiness. To enable YP to assess the trustworthiness and credibility of a public (mental) health resource like an online SSI, those who are advertising the resource should make it immediately clear who the resource is affiliated with (e.g., institutional logos). Information should also be provided at the beginning of the resource about who developed it, alongside links to further plain language summaries regarding development and evaluation for those interested.

It was interesting to note that YP in our study particularly wanted SSIs that gave them information on coping mechanisms and self-compassion. Consistent with this, we also found that frontline non-mental health specialist professionals in the UK thought that online SSIs for YP with depression symptoms should highlight key messages like ‘do more of what matters’ and ‘learn to be kind to yourself’ [56]. Online self-help SSIs are premised on self-determination theory [59], which posits that human motivation is driven by the fulfilment of three basic psychological needs: autonomy, competence, and relatedness. YP’s desire for coping mechanisms may reflect the competence need, as online SSIs are intended to be a source of immediate help that can be accessed autonomously by giving YP evidence-based psychoeducation and helping them, through case examples and action planning, to think about how they can apply this learning. In a UK-based pilot study of an online SSI which combined growth mindset and self-compassion principles [51], analysis of participant responses from a reflective writing task at the end of the SSI found that participants most frequently mentioned acceptance of in-the-moment psychological experiences, followed by self-determination and control. Given that YP are enthusiastic about a self-compassion focused SSI, it may be that Project Care (key message: learn to be kind to yourself), developed and evaluated in the USA [28, 31], could be useful to YP in the UK.

### Strengths and limitations

Strengths of the current study include the large sample of YP, who demonstrate some demographic and clinical diversity, particularly in relation to ethnicity and range of experienced depressive symptoms. The range of depression severity may be because our initial study advertisement mentioned depression, although it was not an eligibility criterion; subsequently, our YP advisors recommended we remove this explicit reference to depression, which we did after ~ 4 weeks, meaning that some of the latter participants might have approached the study from a perspective of wellness.

That said, we only recruited YP who could participate in virtual interviews to allow participation from across the UK, excluding the perspectives of those unable to do so. We also acknowledge that the requirement to turn their camera on for at least some of the interview may have been off-putting for some YP but was a necessity due to incidents of identity fraud. However, we assume that having a YRT member co-conduct the interviews partially mitigated this and made participants more comfortable through shared language and knowledge.

Our sample did not include any 13- or 14-year-olds; as our participants highlighted the importance of age appropriateness, there is a need for future studies to explore how younger adolescents perceive SSIs specifically. Further, the topic guide made use of the *‘Sally’* persona and asked participants to answer the questions with this hypothetical scenario in mind. As such, the findings from this study do not necessarily reflect what YP would do if they were seeking help themselves, which would be interesting to explore in a future study.

### Implications

Contemporary approaches to intervention development center implementation from the outset and throughout the cycle of development [60]. This cycle starts with intervention identification, which in our study was the potential of online self-help SSIs to expand current mental health provision for YP in the UK [18]. The current study has enabled us to consider and refine our ideas about how and where the SSIs we are developing will be signposted to YP, and how they may need to link to other sources of support. This, combined with findings and co-produced guidelines for sharing mental health information for YP online [34], will aid researchers who are developing online interventions within the context of youth mental health, as well as healthcare and frontline professionals looking to find appropriate and trustworthy resources for YP in the early stages of help-seeking.

Retrospectively mapping our findings against the CFIR model highlighted several additional considerations for the implementation of online self-help SSIs for YP. The wider context of media publicity and growing awareness of mental health issues underscores the importance of timely and accessible interventions. Furthermore, the iTHRIVE model [61], which has been widely adopted in CAMHS in the UK, includes the use of self-help and single-session approaches. This suggests that there is potential for SSIs to be integrated into existing service delivery frameworks, although the extent to which this is already happening is unknown. For the individuals involved in receiving help in this single-session format, anonymity and ease of access are crucial for YP, particularly those at early stages of help-seeking or experiencing ambivalence, and this should be prioritized in both research and clinical delivery of these interventions. SSIs can enhance self-efficacy and empowerment, potentially encouraging further help-seeking behaviors. However, self-stigma and mental health symptoms may interfere with the use of SSIs, necessitating careful consideration of user readiness and the fit within service pathways. There is no one size fits all, so ensuring SSIs are not mandated as a step within a stepped care model is vital to avoid potential barriers for some YP in accessing mental health support.

## Conclusion

Overall, our study highlights the largely positive attitudes that YP hold towards online self-help SSIs for mental health, with perceived benefits relating to anonymity, lack of disclosure, immediate and easy accessibility, feelings of autonomy, prevention, and as a gateway to further help-seeking. However, YP also expressed some skepticism, with doubts regarding sufficiency and ability to address more severe mental health problems, alongside logistical considerations relating to where SSIs are advertised, their age appropriateness, and trustworthiness. Going forward, researchers looking to develop online self-help SSIs for YP’s mental health may want to bear some of these considerations in mind, whilst also furthering our understanding by exploring the views of a more diverse range of YP, including those who have utilized SSIs.

## Electronic supplementary material

Below is the link to the electronic supplementary material.

Supplementary Material 1

## Data Availability

The datasets used in the current study may be made available from Dr Maria Loades, data curator, upon reasonable request.
