# Supplementary material for: Young people’s attitudes towards online self-help single-session interventions: findings from a co-produced qualitative study
Source: BMC Psychol. 2025 Apr 24;13:439. doi: 10.1186/s40359-025-02727-8 (PMC12023387; doi:10.1186/s40359-025-02727-8)
Supplement: Supplementary file 1 — Supplementary Material 1 [file 40359_2025_2727_MOESM1_ESM.docx]

**Supplementary Materials**

During the first part of the Can We Connect interview, participants were presented with a persona generated by members of the Young Research Team. This persona describes a young person who is beginning to struggle with low mood and not enjoying things they used to enjoy anymore. The topic guide instructions regarding the use of the persona are displayed in Figure S1, and the questions asked in parts three and four of the interview, which are relevant to the current paper, are displayed in Figure S2.

**Figure S1.** Topic guide instructions regarding the use of the persona, ‘Sally’.

A persona is fictional character.

Here is an example persona I previously generated with PPI input.

*Sally is a 15-year-old girl from a mixed race background. Over the past few months, Sally’s parents and teachers have become increasingly concerned about her low mood and withdrawal from life; she has been less interested than usual in seeing her friends and is reluctant to go out and about with family members, preferring to stay at home. Her form tutor has noticed that she is much more tearful than usual at school, and even relatively minor things, like getting a B grade in a test rather than an A, have been very upsetting to her; she also told her mother that she doesn’t think she is good enough. She continues to go to school, but isn’t doing as well as she usually does, and her grades have slipped. She has opted to give up dancing, which she previously enjoyed.*

The key features we need the persona to display are:

- Starting to struggle with low mood, not enjoying things so much any more. Finding it hard to sleep.
- Doesn't feel ready to talk to anyone yet.
- Suggest keep it simple, don't add too many details that aren't needed.

**Figure S2.** Questions asked in parts three and four of the topic guide, relevant to this paper.

**Interview Part 3**

Now, we would like to show you some information. [Research team member interviewer to screenshare]

1. Examples Instagram advert from Lab for Scalable Mental Health (YES flyer PDF)

- What do you think about this information?
- How trustworthy is it?
- How relevant is it for a young person?
- How credible/believable is it?
- What do you like about it?
- What don’t you like?
- How could this website be improved for young people?

**Interview Part 4**

Remember [our persona]. Imagine they saw an advert on social media with a link to help. This help was doing some activities online, as a one-off, and would take about 20-30 minutes. The activities would help them to learn to be kinder to themselves. They don’t have to talk to anyone or ask for permission to use it. We call these ‘single session interventions’.

- How do you think they would feel about this option?
- What might get in the way of them doing this?
- What could be good about this kind of offer?
- What kinds of topics might be useful?
